# Special Issue “Effects of Dyslipidemia and Metabolic Syndrome on Cardiac and Vascular Dysfunction”

**DOI:** 10.3390/ijms26010155

**Published:** 2024-12-27

**Authors:** Isotta Chimenti, Vittoria Cammisotto

**Affiliations:** 1 Department of Medical-Surgical Sciences and Biotechnologies, Sapienza University of Rome, 04100 Latina, Italy; 2 Maria Cecilia Hospital, GVM Care & Research, 48033 Cotignola, Italy; 3 Department of Clinical Internal, Anesthesiological and Cardiovascular Sciences, Sapienza University of Rome, Viale del Policlinico 155, 00161 Rome, Italy; vittoria.cammisotto@uniroma1.it

## Introduction

The global increase in dysmetabolic conditions such as hyperglycemia, insulin resistance, dyslipidemia, metabolic syndrome, and type 2 diabetes is becoming a significant healthcare concern. These conditions are major contributors to the incidence of cardiovascular diseases (CVDs), including atherosclerosis and different cardiomyopathies [1], which represent significant health problems. Despite continuous scientific progress in understanding these disorders and in the development of potential new treatments, the complex metabolic and molecular mechanisms that drive their progression remain only partially understood. Among them, dyslipidemia plays a particularly fundamental role in the etiology and development of CVDs, especially in individuals with metabolic syndrome [2].

Dyslipidemia in metabolic syndrome is characterized by elevated circulating levels of triglycerides and apolipoprotein-rich particles, along with small low-density lipoprotein cholesterol (LDL-C) and reduced levels of high-density lipoprotein cholesterol (HDL-C). This lipid imbalance is a major factor in the formation and progression of atherosclerotic plaques, which gradually impair vascular function [3]. Moreover, dyslipidemia plays a key role in the progression of cardiac dysfunction by promoting lipid accumulation inside cardiomyocytes, disrupting their mitochondrial metabolism, and altering cellular signaling pathways that regulate healthy myocardial and vascular functions [4]. Many studies have shown how dyslipidemia and metabolic syndrome affect multiple metabolic processes, including fatty acid oxidation and insulin signaling, as well as cytoprotective pathways such as autophagy [5,6]. Their disruption leads to oxidative stress, inflammation, and excessive inflammatory responses, further worsening the cardiovascular damage. Understanding the cellular and molecular mechanisms underneath all these signals may offer hope for identifying new therapeutic targets for both CVD prevention and treatment in people who are at risk of being affected by dyslipidemia and metabolic syndrome.

The articles collected in this Special Issue provide novel insights into multiple pathogenetic connections and mechanisms. The first study by Szekeres R et al. [7] explored the potential benefits of *Prunus cerasus* (sour cherry) extract in a rabbit model of atherosclerosis-induced diastolic dysfunction. The findings revealed that this anthocyanin-rich extract improved both lipid profiles and cardiac function, increasing the myocardial levels of protective markers such as endothelial nitric oxide synthase (eNOS), protein kinase G (PKG), and sarcoplasmic reticulum Ca^2+^-APTase (SERCA2a), while reducing oxidative stress markers. This suggests that sour cherry extract could be a promising therapeutic option or additive for managing atherosclerosis-related cardiac dysfunction [7].

Another study in our Special Issue, conducted by Marino F et al. [8], compared the effects of streptozotocin (STZ)-induced type 1 and type 2 diabetes mellitus (T1DM and T2DM) on diabetic cardiomyopathy (DCM) **in C57BL/6J mice**. The authors found that the induction of a T1DM-like condition led to more severe cardiac dysfunction than the T2DM-like model, with heightened indexes of oxidative stress, apoptosis, hypertrophy, and fibrosis. Furthermore, transcriptomic analyses revealed significant differences between the two models, highlighting their respective usefulness and the need to choose appropriate models for studying different aspects of diabetic cardiomyopathy [8].

A related review by Katsi V et al. [9] discussed the shared molecular pathways between atherosclerosis, diabetes, and cancer. This review highlighted how many mechanisms relating to dyslipidemia and endothelial dysfunction in atherosclerosis interestingly overlap with some of the molecular processes that are involved in cancer development. This suggests that the metabolic disturbances seen in conditions such as metabolic syndrome could also play a role in cancer progression, opening up new perspectives for the clinical approach to and therapeutic interventions for these diseases [9].

Another interesting article by Christou MA et al. [10] discussed the effects of hypoglycemia on cardiovascular function in diabetic patients. Hypoglycemia is a common occurrence in diabetic individuals who are treated with insulin or sulfonylureas and has been linked to various CVDs and related risks, including arrhythmias, endothelial dysfunction, and increased oxidative stress. Although there is strong evidence associating hypoglycemia with an elevated CVD risk, the causal relationship is not fully understood. Fortunately, recent antidiabetic therapies and advances in monitoring technologies (such as continuous glucose monitoring) are helping to delay the onset of hypoglycemia and its cardiovascular consequences [10].

Finally, a comprehensive review by Puddu A et al. [11] on caveolin-1 collected all the recent insights on its role in atherosclerosis. In fact, caveolin-1 is known to regulate LDL metabolism inside endothelial cells, although it could play either a pro- or anti-atherogenic role depending on the microenvironment. This review also recognized caveolin-1 as a critical player in lipid metabolism and vascular inflammation but still suggested that it could be a key target for future atherosclerosis treatments [11].

Collectively, the articles in this Special Issue have contributed to expanding our understanding and thoughts on how dyslipidemia and metabolic syndrome contribute to cardiovascular dysfunction and CVDs. By identifying new molecular and metabolic pathways, as well as by identifying novel interactions among known players that are involved in these conditions, they offer new perspectives onr the development of therapeutic strategies that could help reduce the burden of CVDs in patients with metabolic disorders.

## Data Availability

No new data were created.
